# Supplementary material for: Dynamic Compression Flows for Neuroscience Data
Source: bioRxiv. 2026 Feb 13:2026.02.12.705535. Preprint. [Version 1] doi: 10.64898/2026.02.12.705535 (PMC12919234; doi:10.64898/2026.02.12.705535)
Supplement: Supplement 2 [file NIHPP2026.02.12.705535v1-supplement-2.pdf]

bioRxiv preprint doi: https://doi.org/10.64898/2026.02.12.705535; this version posted February 13, 2026. The copyright holder for this preprint (which was not certified by peer review) is the author/funder, who has granted bioRxiv a license to display the preprint in perpetuity. It is made available under aCC-BY 4.0 International license.

A. Additional Results for Ball Video Data

This appendix reports additional ablations for the rotating-ball experiment in Section 4.1.

A.1. Regularization on D

We study shrinkage regularization on $\mathbf{D} = \mathrm{diag}(d_1, \ldots, d_D)$, which controls the per-coordinate contribution in the compressed endpoint $\mathbf{x}^{(0)} = \mathbf{b} + \mathbf{L}\mathbf{D}^{1/2}\boldsymbol{\mu}_{\psi}(\mathbf{x})$ when $\mathbf{L}$ is orthonormal and $\boldsymbol{\mu}_{\psi}$ is normalized. In addition to ridge and LASSO, we consider the horseshoe penalty, a global-local shrinkage prior that strongly suppresses small coordinates while leaving large d_i nearly unchanged due to its heavy tails (Carvalho et al., 2010). Figure S1 summarizes how different penalties reshape the learned scale profile $\{\sqrt{d_i}\}$ while preserving the dominant 3D latent geometry and projected velocity structure.

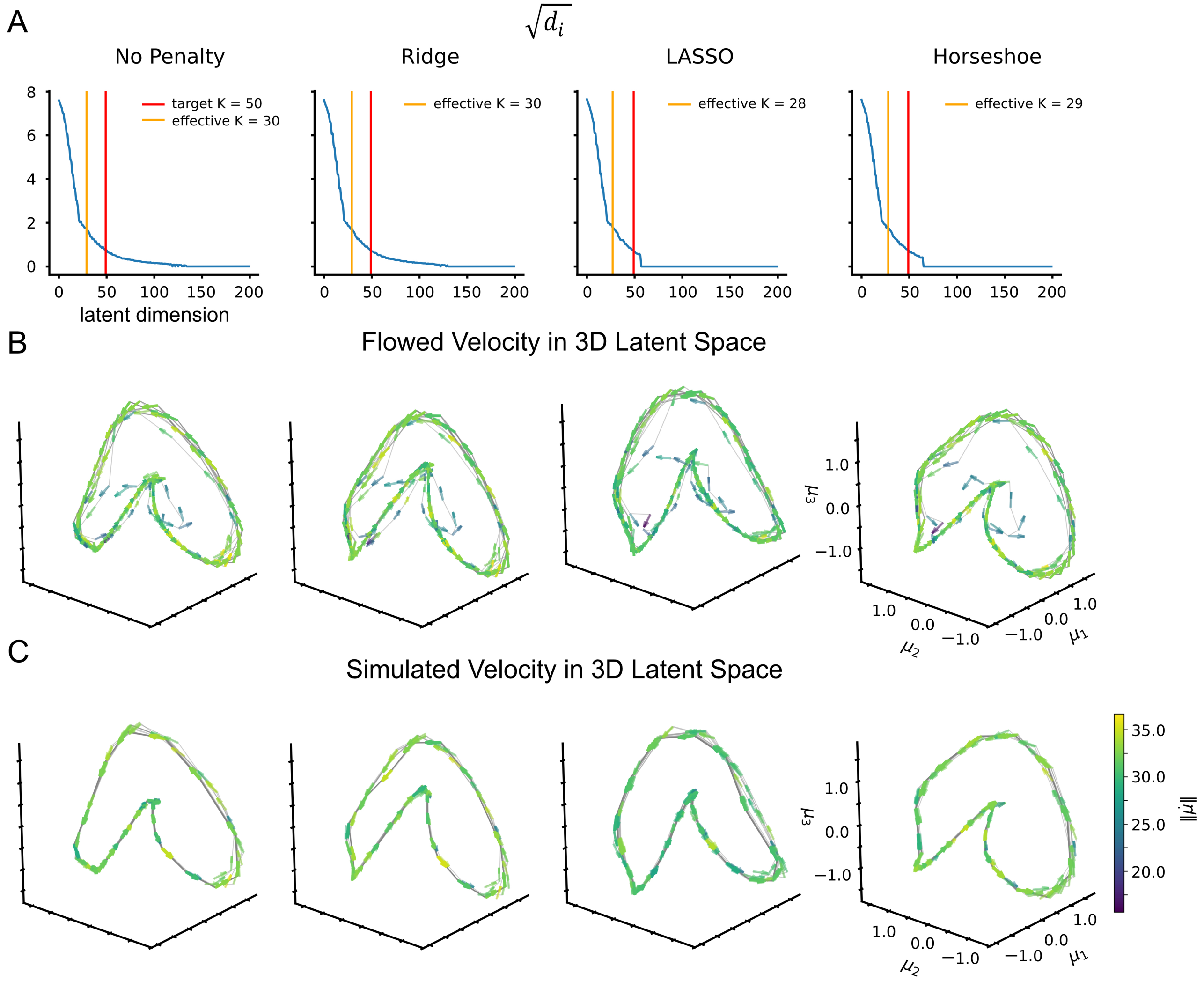

Figure S1. **Effect of shrinkage penalties on D for ball simulation.** Same setting as **Figure 3** ($p = 1/50$, $h = 0$), but with different penalties on the diagonal scales $\{d_i\}$: none, ridge, LASSO, and horseshoe (each with a different random seed). (**A**) Learned $\sqrt{d_i}$ as a function of latent index. With orthonormal $\mathbf{L}$ and normalized $\boldsymbol{\mu}_{\psi}$ (Section 2.2), larger d_i indicates higher contribution of dimension i. The red line marks $K_{\text{target}} = \mathbb{E}(K) = 50$, and the orange line marks the effective dimension K_{eff} (smallest K explaining 95% of $\sum_i d_i$). (**B**) Projected dynamical velocities in the first three latent coordinates for *flowed* trajectories $\tilde{\mathbf{x}}_t^{(0)}$. (**C**) Projected dynamical velocities for *simulated* trajectories (integrating $\tilde{\mathbf{x}}_0^{(0)}$ forward using $\mathbf{v}_{\boldsymbol{\theta}}^{(0)}$). Arrows are normalized to unit length to emphasize direction, and color indicates the velocity magnitude $\|\dot{\tilde{\mathbf{x}}}_t^{(0)}\|_2$ in the full ambient space. Across penalties, the latent geometry and velocity fields are consistent.

bioRxiv preprint doi: https://doi.org/10.64898/2026.02.12.705535; this version posted February 13, 2026. The copyright holder for this preprint (which was not certified by peer review) is the author/funder, who has granted bioRxiv a license to display the preprint in perpetuity. It is made available under aCC-BY 4.0 International license.

A.2. Soft 3D latent space

Figure S2 shows results for experiments using a much tighter nested-dropout budget ($p = 1/3$, so $\mathbb{E}(K) = 3$), showing that the same loop-shaped latent trajectories are retained under a soft 3D representation. Additionally, we compare simulated frames at $\tau = 0$ obtained from either encoder- or flow-based ($\boldsymbol{u}_{\boldsymbol{\phi}}^{(\tau)}$) compression (**Figure S2A**).

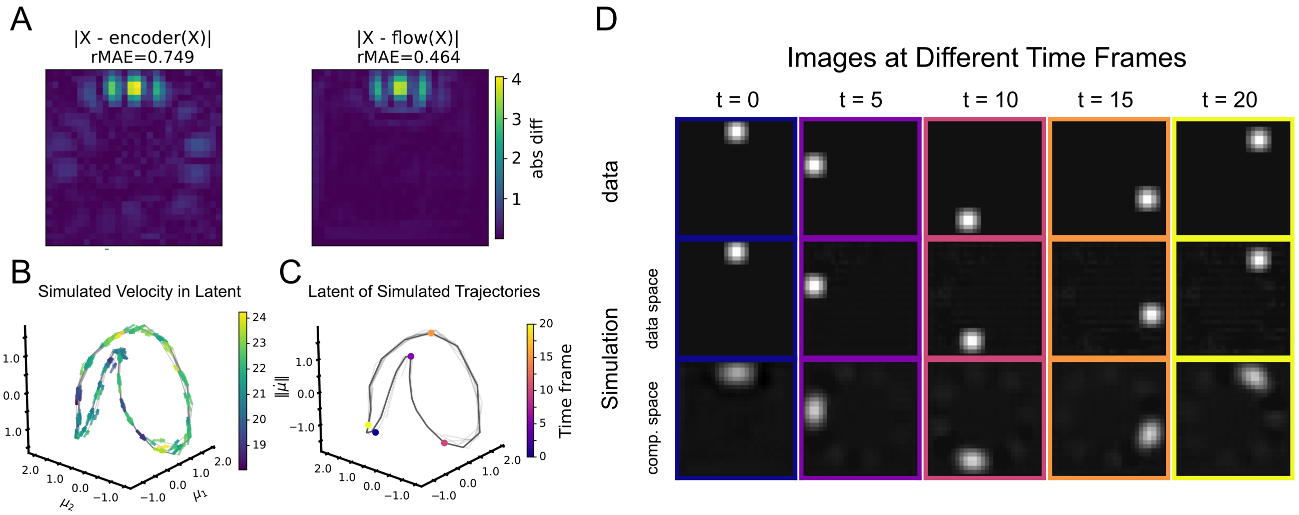

Figure S2. **Soft 3D latent representation via nested dropout for ball simulation.** We repeat the rotating-ball experiment with no history ($h = 0$) and a tighter nested-dropout budget $p = 1/3$ (so $K_{\text{target}} = \mathbb{E}(K) = 3$). (**A**) Absolute per-pixel deviation between the original frame and its compression to $\tau = 0$, comparing the encoder endpoint versus the learned compressive flow (rMAE: root mean absolute error per pixel). (**B**) Projected dynamical velocities from *simulated* trajectories in the first three latent coordinates. (**C**) Simulated trajectories in the same 3D latent space, with five representative time points from trial 1 highlighted. (**D**) Corresponding ground-truth frames (top), rollouts in data space (middle, $\tau = 1$), and rollouts in compressed space (bottom, $\tau = 0$).

bioRxiv preprint doi: https://doi.org/10.64898/2026.02.12.705535; this version posted February 13, 2026. The copyright holder for this preprint (which was not certified by peer review) is the author/funder, who has granted bioRxiv a license to display the preprint in perpetuity. It is made available under aCC-BY 4.0 International license.

A.3. Hyperparameter settings for loss terms

In the main text, we use $\alpha = \beta = \eta = 1$ for all experiments. Here, we show that the results, including latent geometry, are robust under different loss weights, i.e., $(\alpha, \beta, \eta) = (5, 1, 1)$, $(1, 5, 1)$, and $(1, 1, 5)$.

We study sensitivity to the loss weights in (9). Using the same rotating-ball setting as Section 4.1 ($p = 1/50$, $h = 0$ and no penlaty of $\mathbf{D}$), we retrain DCF while increasing one weight by $5\times$ and keeping the other two fixed: $(\alpha, \beta, \eta) \in \{(5, 1, 1), (1, 5, 1), (1, 1, 5)\}$. Figure S3 compares the learned scale profile ($\sqrt{d_i}$) and the projected 3D latent velocities for both flowed and simulated trajectories.

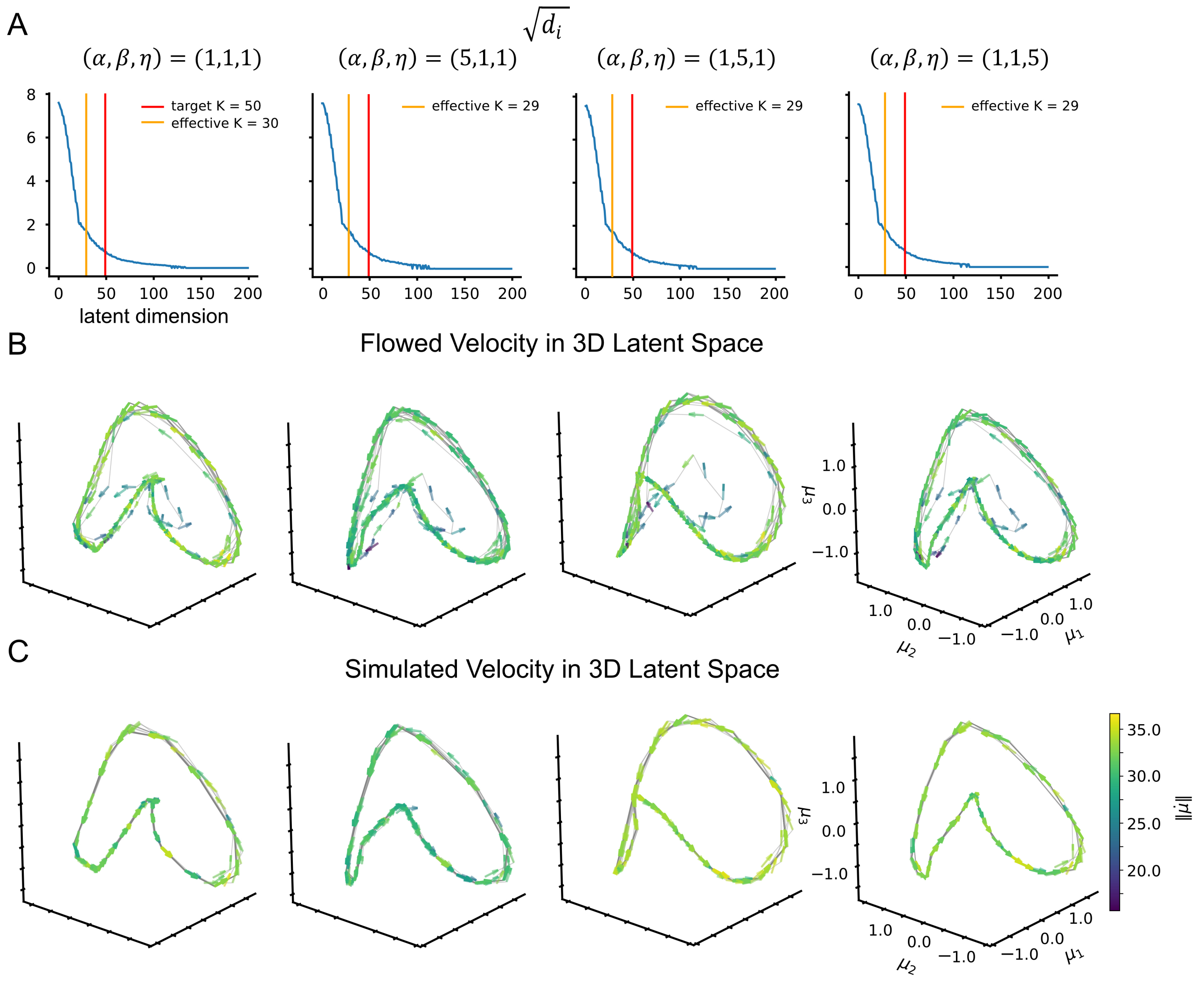

Figure S3. **Sensitivity to loss weights (rotating-ball simulation).** Same setting as Section 4.1 ($p = 1/50$, $h = 0$ and no penalty on $\mathbf{D}$), retrained with $(\alpha, \beta, \eta) \in \{(1, 1, 1), (5, 1, 1), (1, 5, 1), (1, 1, 5)\}$. (**A**) Learned scale profile $\{\sqrt{d_i}\}$ versus latent index. The red line marks $K_{\text{target}} = \mathbb{E}(K) = 50$, and the orange line marks the effective dimension K_{eff} (smallest K explaining 95% of $\sum_i d_i$). (**B**) Projected dynamical velocities for *flowed* trajectories in the first three latent coordinates. (**C**) Projected dynamical velocities for *simulated* trajectories. Arrows are normalized to unit length to emphasize direction, and color indicates velocity magnitude.

bioRxiv preprint doi: https://doi.org/10.64898/2026.02.12.705535; this version posted February 13, 2026. The copyright holder for this preprint (which was not certified by peer review) is the author/funder, who has granted bioRxiv a license to display the preprint in perpetuity. It is made available under aCC-BY 4.0 International license.

A.4. Latent spaces for comparison models on the balls toy dataset

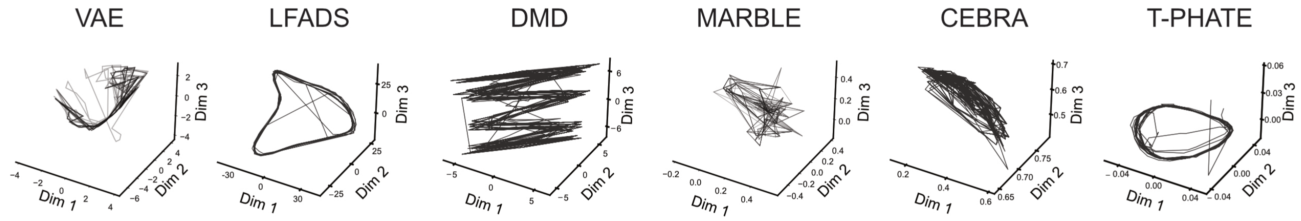

Figure S4. **3D latent representations of comparison models on the rotating ball dataset** Nearly all comparison models fail to capture the cyclical latent structure of the ball dataset; those that do display more variable latent trajectories than DCF.

B. Training Time and Experiment Parameters

Dataset	K	Lag	Penalty	Total Train Time (Mimgs)	Total Train Time (hours)
Ball	50	0	None	4.27	1.97
	50	0	Ridge	4.27	1.97
	50	0	Lasso	4.27	1.97
	50	0	Horseshoe	4.27	1.97
	3	0	None	4.27	1.17
Maze	50	10	None	55.70	8.51
Mouse	50	0	None	20.82	5.7
Bird	50	0	None	32.61	8.15

Table S1. **Experiment training times and parameters.** Total training times, measured in both 10^6 images (i.e., Mimgs) and hours across our different simulated and neuroscience data experiments. All experiments were conducted on single NVIDIA RTX 4090 GPUs, with per-GPU batch size of 16. K_{target} specifies the geometric distribution used with nested dropout, and lag measures the size of the history window used for contextual prediction (i.e., $\mathbf{x}_{\text{hist}}^{(\tau)}$).

C. Trajectory roll-out times

Dataset	K	Lag	Total Trials	Trial Length	Total Roll-Out Time ($\tau = 0$)	Total Roll-Out Time ($\tau = 1$)
Ball	50	0	10	50	2s	2s
	3	0	10	50	2s	2s
Maze	50	10	592	100	4s	5s
Mouse	50	0	1	1438	1.45min	2min
Bird	50	0	262	26	13s	13s

Table S2. **Trajectory Roll-Out Times.** Roll-out times at $\tau = 0$ and $\tau = 1$ for the different experiments performed. Roll-out simulations were conducted using single NVIDIA RTX 4090 GPUs, and trials were stacked. All values included here are for experiments without added penalties on $\mathbf{D}$. Use of additional regularizers on $\mathbf{D}$ yields comparable roll-out times.

bioRxiv preprint doi: https://doi.org/10.64898/2026.02.12.705535; this version posted February 13, 2026. The copyright holder for this preprint (which was not certified by peer review) is the author/funder, who has granted bioRxiv a license to display the preprint in perpetuity. It is made available under aCC-BY 4.0 International license.

D. Additional Experiments for Monkey Center-Out Reach (Maze) Data

This appendix reports results obtained on neural center-reach out (maze) data. **Table S3** shows that our model easily outperforms all other competing (generative) approaches in reconstructing firing rates on held-out (test) data. **Figure S5** shows that our model correctly infers topographically organized trajectories corresponding to the different (center-reach) directions used throughout the experiment. Flowed trajectories for our model were obtained by compressing the observed data $\mathbf{x}_t^{(1)}$ pointwise to latent space ($\tau = 0$) by integrating the compressive flow $\mathbf{u}_\phi$ at each t.

Method	Median R^2 $(25^{th}\text{percentile}, 75^{th}\text{percentile})$
**DCF (ours)**	$0.999\ (0.998, 0.999)$
VAE	$0.618\ (0.559, 0.678)$
LFADS	$0.595\ (0.527, 0.661)$

Table S3. **Model comparisons: neural data.** Reconstruction of neural activity (firing rates) on held out test-data. Reported values are R^2 quartiles across held-out trials.

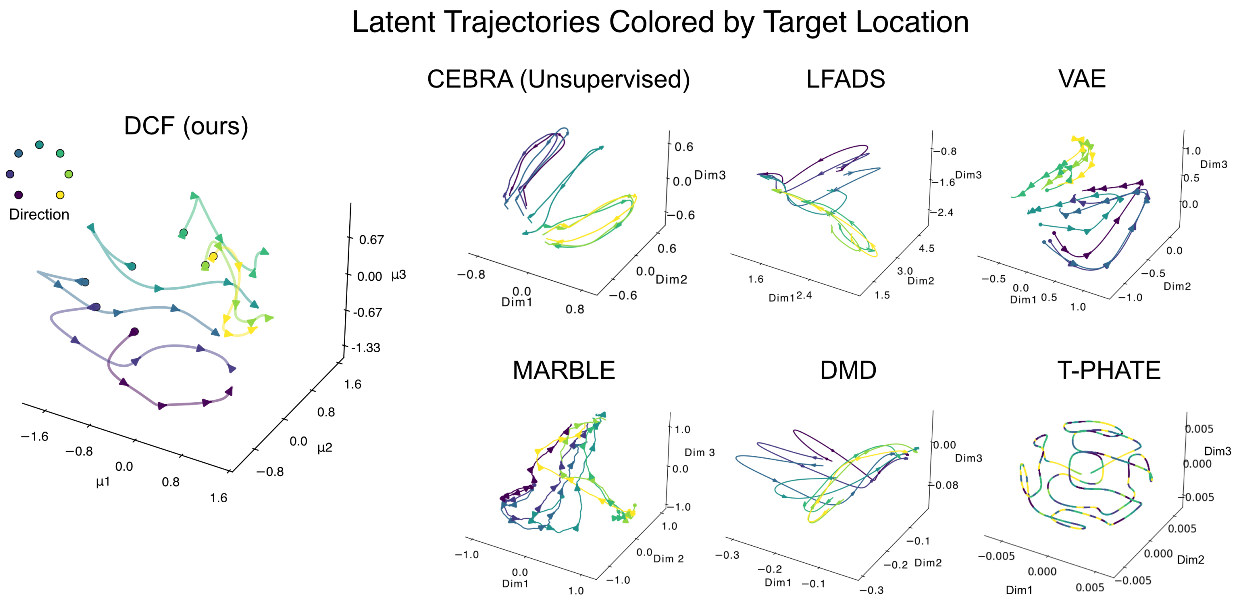

Figure S5. **3D latent representations of neural data. Left:** Flowed mean trajectories (compressing data pointwise via compressive flow $\mathbf{u}_\phi$) in the first three latent coordinates. Color indicates monkey reach direction; lines are averaged across reach direction. **Right:** 3D latent trajectories of comparison models. The latent representation of CEBRA (without supervision), LFADS, VAE, MARBLE and DMD are averaged across monkey reach location. T-PHATE latent representation is unstructued and therefore not averaged.

bioRxiv preprint doi: https://doi.org/10.64898/2026.02.12.705535; this version posted February 13, 2026. The copyright holder for this preprint (which was not certified by peer review) is the author/funder, who has granted bioRxiv a license to display the preprint in perpetuity. It is made available under aCC-BY 4.0 International license.

E. Additional experiments on mouse video data

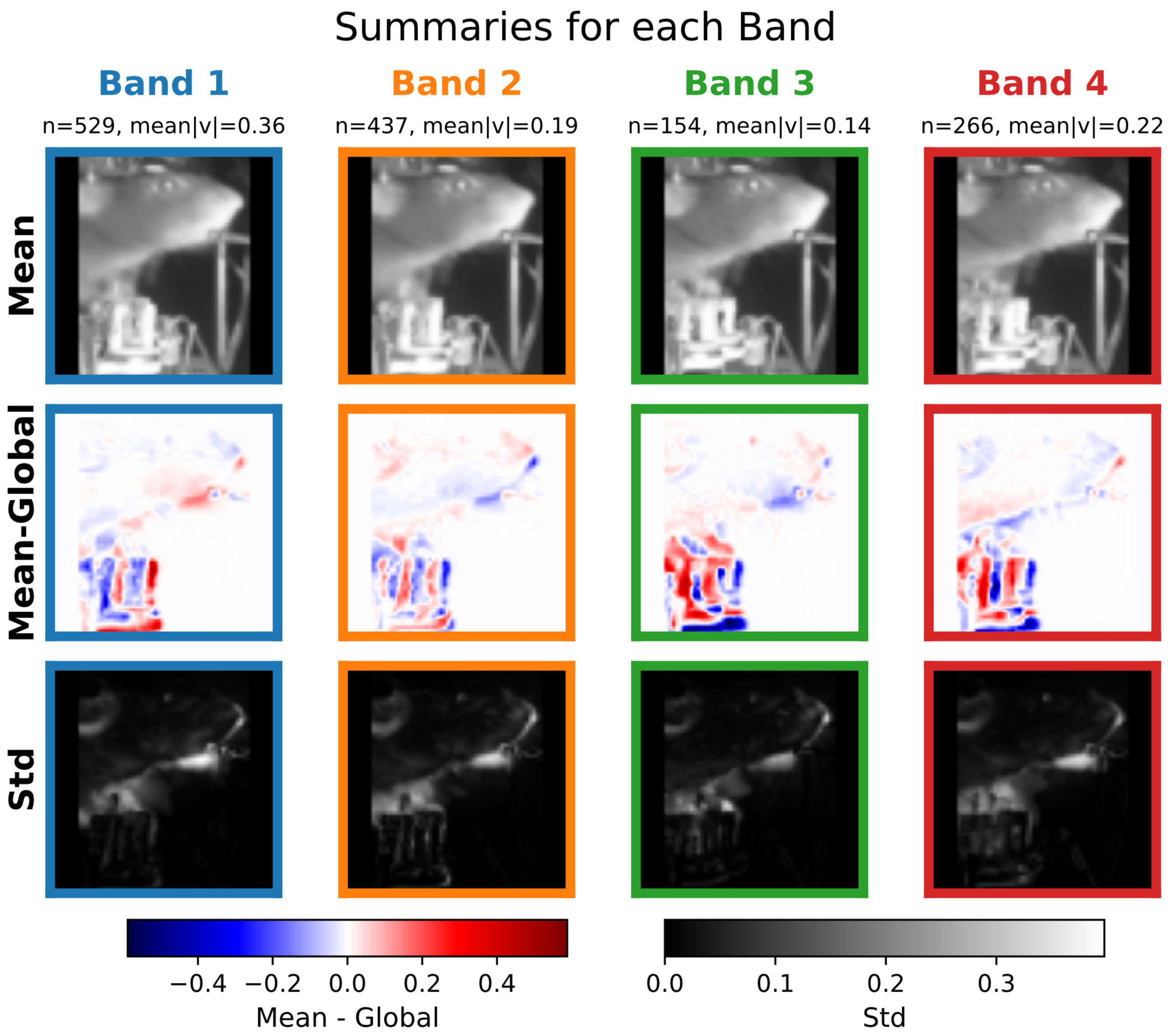

Figure S6. **Band-level summaries for mouse video data.** For each band in **Figure 4A**, we show the mean frame (top), the mean deviation from the global mean (middle, mean minus global), and the per-pixel standard deviation (bottom). We report the number of frames n and the mean velocity magnitude within each band. Of note, moving top to bottom across bands changes video from periods of small, more quiescent activity to periods of larger paw and mouth/tongue movement. In general, our blue band captures mostly wider mouth and tongue movements (i.e., repetitive licking), whereas our orange, red, and green bands capture a mix of both paw and mouth/tongue movement, with movements becoming more subtle as we move from orange $\rightarrow$ red $\rightarrow$ green. We include a supplemental video (`Latent_Plot_DCF.mp4`) showcasing these frame transitions as we move in our inferred latent space.

bioRxiv preprint doi: https://doi.org/10.64898/2026.02.12.705535; this version posted February 13, 2026. The copyright holder for this preprint (which was not certified by peer review) is the author/funder, who has granted bioRxiv a license to display the preprint in perpetuity. It is made available under aCC-BY 4.0 International license.

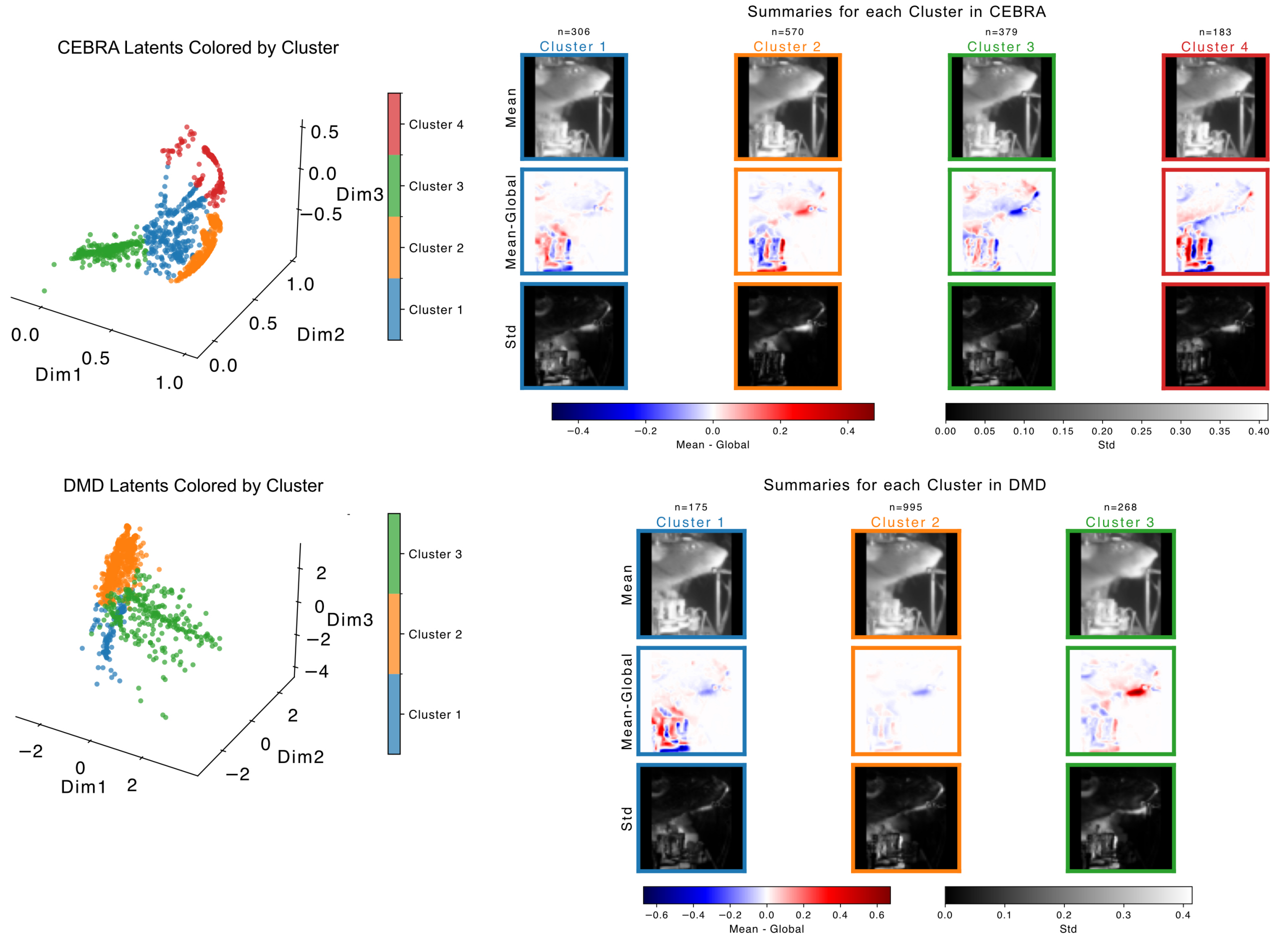

Figure S7. **Latent space structure in DMD and CEBRA comparison models.** Latent spaces (left columns, color-coded by clusters) and cluster mean frame, mean deviation from global mean, and per pixel standard deviation (right columns, same construction as in **Figure S6**). In general, both DMD and CEBRA fail to separate outliers related to brisk controller movements from rest of data. Additionally, clusters identified often mix periods of quiescent activity with movement and mix more than one type of movement within the same cluster, providing overall less structured latent spaces. We include supplemental videos `Latent_Plot_DMD.mp4` and `Latent_Plot_CEBRA.mp4` showing frame transitions as we move through the latent spaces inferred by these competing models.

bioRxiv preprint doi: https://doi.org/10.64898/2026.02.12.705535; this version posted February 13, 2026. The copyright holder for this preprint (which was not certified by peer review) is the author/funder, who has granted bioRxiv a license to display the preprint in perpetuity. It is made available under aCC-BY 4.0 International license.

F. Additional experiments on bird audio data

Dataset (raw audio and labeled syllables) and accompanying pre-processing code (`get_specs.py`) for bird audio experiments is included in **Supplements** under `bird_data`. Raw audio data are included as `.wav` files inside the `audio` folder, under subdirectories named by date of data collection (YYYYMMDD format) and syllable labels are included as `.txt` files under the `labeled_syllables` folder, with similar date-named subdirectory structure.

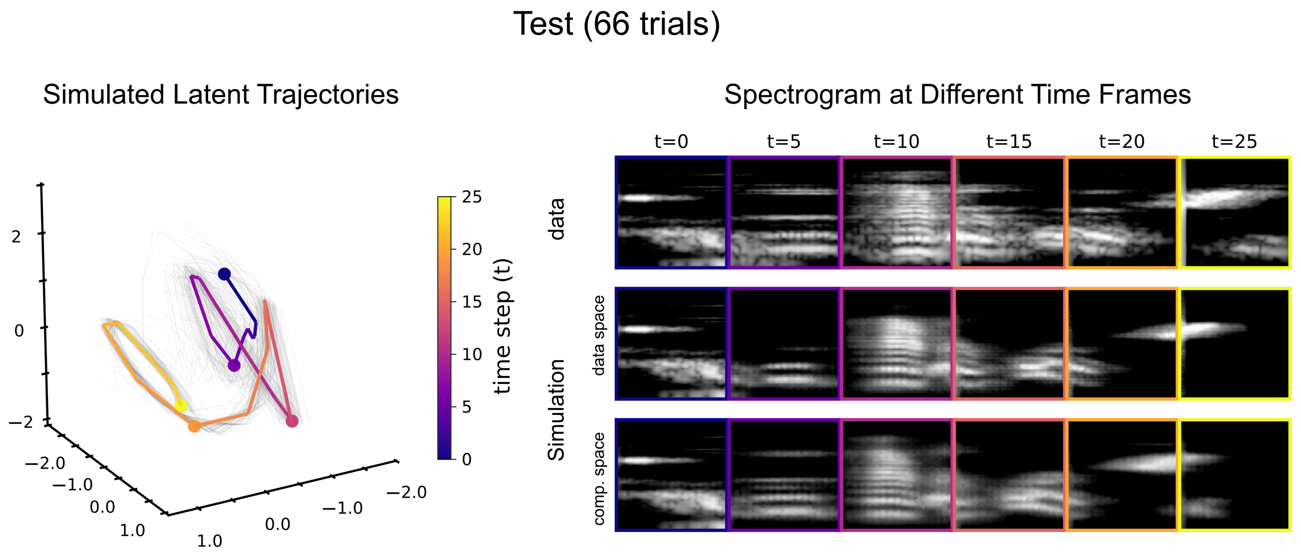

Figure S8. **Birdsong rollouts on the test set.** Left: simulated latent trajectories in the first three coordinates of $\tilde{\boldsymbol{\mu}}$ for held-out trials. Right: ground-truth spectrogram frames (top) and corresponding simulated rollouts decoded in data space (middle, $\tau = 1$) and compressed space (bottom, $\tau = 0$), shown at matched time points.

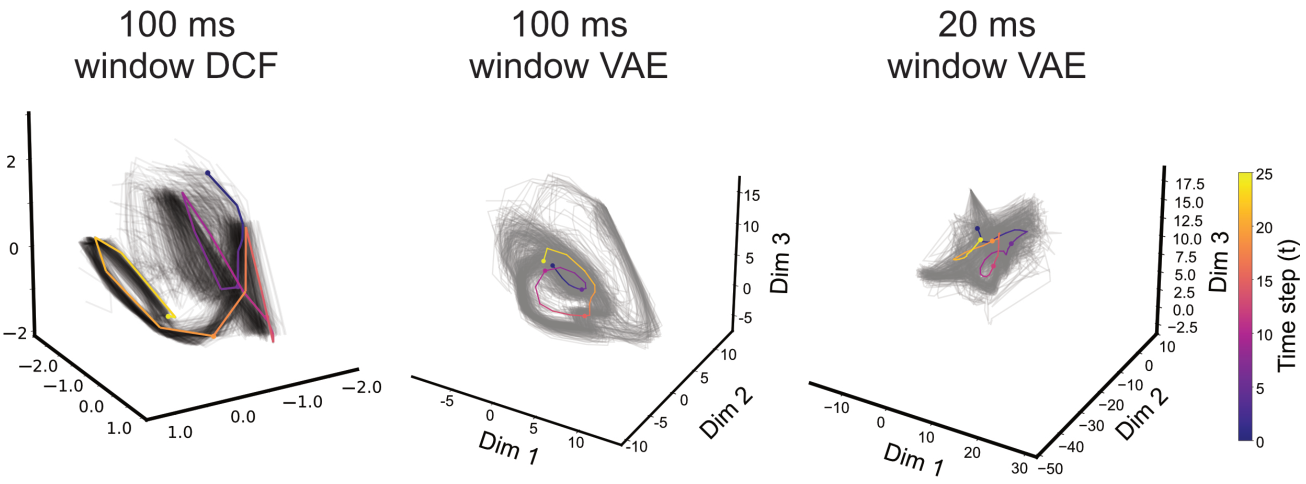

Figure S9. **Comparison embeddings using a 3d VAE.** Left: simulated trajectories from a DCF model (cp. **Figure 5C**). Middle: embeddings of a VAE trained using 100 ms long spectrogram windows. Right: embeddings of a VAE trained using 20ms long spectrogram windows. In general, VAEs with short data windows produce latent spaces with disorganized temporal structure, while longer data windows exhibit more obvious structure but more variability than DCF.
